# Crystal structure and functional implications of the tandem-type universal stress protein UspE from *Escherichia coli*

**DOI:** 10.1186/s12900-016-0053-9

**Published:** 2016-02-11

**Authors:** Yongbin Xu, Jianyun Guo, Xiaoling Jin, Jin-Sik Kim, Ying Ji, Shengdi Fan, Nam-Chul Ha, Chun-Shan Quan

**Affiliations:** Department of Bioengineering, College of Life Science, Dalian Nationalities University, Dalian, 116600 Liaoning China; Laboratory of Biomedical Material Engineering, Dalian Institute of Chemical Physics, Chinese Academy of Sciences, Dalian, 116023 Liaoning China; Department of Agricultural Biotechnology, College of Agriculture and Life Sciences, Seoul National University, Gwanak-gu, Seoul 151-742 Republic of Korea

**Keywords:** UspE, UspA superfamily, Tandem-type USP

## Abstract

**Background:**

The universal stress proteins (USP) family member UspE is a tandem-type USP that consists of two Usp domains. The UspE expression levels of the *Escherichia coli* (*E. coli*) become elevated in response to oxidative stress and DNA damaging agents, including exposure to mitomycin C, cadmium, and hydrogen peroxide. It has been shown that UspA family members are survival factors during cellular growth arrest. The structures and functions of the UspA family members control the growth of *E. coli* in animal hosts. While several UspA family members have known structures, the structure of *E. coli* UspE remains to be elucidated.

**Results:**

To understand the biochemical function of UspE, we have determined the crystal structure of *E. coli* UspE at 3.2 Å resolution. The asymmetric unit contains two protomers related by a non-crystallographic symmetry, and each protomer contains two tandem Usp domains. The crystal structure shows that UspE is folded into a fan-shaped structure similar to that of the tandem-type Usp protein PMI1202 from *Proteus mirabilis*, and it has a hydrophobic cavity that binds its ligand. Structural analysis revealed that *E. coli* UspE has two metal ion binding sites, and isothermal titration calorimetry suggested the presence of two Cd^2+^ binding sites with a K_d_ value of 38.3–242.7 μM. Structural analysis suggested that *E. coli* UspE has two Cd^2+^ binding sites (Site I: His117, His 119; Site II: His193, His244).

**Conclusion:**

The results show that the UspE structure has a hydrophobic pocket. This pocket is strongly bound to an unidentified ligand. Combined with a previous study, the ligand is probably related to an intermediate in lipid A biosynthesis. Subsequently, sequence analysis found that UspE has an ATP binding motif (Gly^269^- X_2_-Gly^272^-X_9_-Gly^282^-Asn) in its C-terminal domain, which was confirmed by *in vitro* ATPase activity monitored using Kinase-Glo® Luminescent Kinase Assay. However, the residues constituting this motif were disordered in the crystal structure, reflecting their intrinsic flexibility. ITC experiments revealed that the UspE probably has two Cd^2+^ binding sites. The His117, His 119, His193, and His244 residues within the β-barrel domain are necessary for Cd^2+^ binding to UspE protein. As mentioned above, USPs are associated with several functions, such as cadmium binding, ATPase function, and involvement in lipid A biosynthesis by some unknown way.

## Background

The universal stress proteins (USP) superfamily is a group of conserved proteins that play an important role in *E. coli.* USPs’ expression levels become elevated in response to a bewildering variety of stress conditions, such as heat shock, nutrient starvation, the presence of oxidants, DNA-damaging agents (including exposure to mitomycin C, cadmium, and hydrogen peroxide), as well as others, that may arrest cell growth. Proteins in the UspA family constitute a natural biological defense mechanism [1, 2]. Despite considerable research on the behavior of UspA family members, the biological and biochemical roles of these proteins remain largely uncharacterized. Very few details were available to help decipher their roles in the aforementioned cellular processes [1]. A better understanding of the molecular mechanisms of *E. coli’s* UspA proteins is important for establishing effective therapeutic strategies. In particular, establishing the three-dimensional structural model of the UspE protein can provide hints to explore the function(s) of the UspA family.

*E. coli* has six small UspA superfamily genes*: uspA*, *-C -D*, *-E*, *-F*, and *-G*. To date, these proteins have been extensively investigated. Previous studies have shown that UspA family members show immaculate similarity. They encode either a small USP protein (approximately 14 to 15 kDa) that consists of two USP domains in tandem or a larger version (approximately 30 kDa) that consists of two peptides attached as a single functional protein [3, 4]. UspA, UspC, and UspD belong to class I; UspF and UspG belong to class II; two Usp domains of UspE belong to class II and IV based on the sequence and structural analysis [3, 5, 6]. Previous works have found that while Usp family members have partially overlapping functions, the functions of class I, II, and IV Usps are distinct [7]. UspA proteins differ in their responses to protect cells from oxidative stress and DNA damage agents; UspA, UspC, UspD, and UspE are induced by exposure to mitomycin C, cadmium, and hydrogen peroxide. However, class II proteins, UspG and UspF, were associated with iron scavenging in the cell [4]. As mentioned before, UspE is a tandem-type USP. When UspE proteins are split apart and treated separately, the UspE2 domain is more closely related to UspF and UspG. This is clearly visible in both the clustering analysis and the reconstructed cladogram. In contrast, UspE1 groups are more closely related to class I UspA proteins (UspACD) [1].

This paper includes structural and functional studies on UspE from *E. coli*. Specifically, it presents the three-dimensional X-ray crystal structure of the recombinantly produced UspE from *E. coli* at 3.2 Å resolution. Additionally, through the use of structural biochemical analyses, the UspE mechanisms were determined. In terms of its overall structure, UspE was found to be similar to the tandem-type Usp protein PMl1202 from *Proteus mirabilis*, which has a hydrophobic cavity that binds an unidentified ligand. It was also observed that UspE has an ATP binding motif (Gly^269^-Thr-Val-Gly^272^-X_9_-Gly^282^-Asn) in its C-terminal domain. The ATPase activity was then measured to determine if UspE had ATPase activity and to characterize UspE activity. Because previous research found that UspE is critical for Cd^2+^ defense, we characterized the role of UspE as part of the Cd^2+^ binding process by ITC and structural analysis and found that UspE has two Cd^2+^ binding sites in its tandem USP domain. These observations suggest that UspE performs several distinct functions, such as ATP hydrolysis and cadmium defense. Although the molecular function of this protein remains unknown, our three-dimensional structures of UspE offer valuable clues to understand its potential biochemical mechanisms.

## Methods

### Structure determination, refinement and protein data bank accession number of UspE

We have previously reported the crystallization and preliminary X-ray analysis of *E. coli* UspE [8]. Data collection and refinement statistics are summarized in Table 1. The structure of the *E. coli* UspE protein was determined by molecular replacement (MR) using the program CCP4 package [9]. We used the coordinates of the structures of *P. mirabilis* PMl1202 (78.5 % sequence identity; PDB code: 3OLQ) as a reference. To build our protein model, we first removed model bias by rounds of simulated annealing performed with the program PHENIX [10], followed by calculating the differences using Fourier maps. Then, the UspE model was rebuilt in the graphic program COOT [11]. The model was finally refined using the same programs by iterative rounds of energy minimization, B-factor, and anisotropic refinements. Then, the composite omit and differences were calculated by Fourier maps. UspE coordinates and structure have been deposited in the Protein Data Bank [12] under accession code: 5CB0 (www.rcsb.org/pdb).

**Table 1:** Diffraction statistics

X-ray source	Beamline 5C, Pohang Accelerator Laboratory
PDB code	5CB0
Wavelength (Å)	1.000
Space group	*I*4_1_22
Resolution (Å)	19.9-3.2
Parameters (Å)	*a* = *b* = 121.1 Å, *c* = 241.7 Å, *α* = *β* = *γ* = 90^o^
Rsym (%)	15.3 % (33.4 %)
Completeness (%)	92.7 (90.2)
Redundancy	5.3 (3.6)
Average *I/σ (I)*	9.2 (3.3)
R-factor (%)	24.31
Rfree (%)	30.06
Rmsd for bonds (Å)	0.007
Rmsd for angles (◦)	1.393
Ramachandran plot (%)	
Favored regions	93.64 %
Allowed regions	5.12 %
Disallowed regions	1.24 %
Number of atoms	
Protein	4630
Ligand	34
Average B factor (Å^2^)	70.54

†*R*_merge_=∑_*hkl*_∑_*i*_│ *I*_*i*_ *(hkl)-<I(hkl) >*│∑_*hkl*_∑_*i*_│ *I*_*i*_ *(hkl)* , where I(hkl), where I(hkl) is the intensity of reflection *hkl*, ∑_*hkl*_ is the sum over all reflections and ∑_*I*_ is the sum over *i* measurements of reflection *hkl*. ‡*R*_work_ =∑_*hkl*_│*F*_o_-*F*_c_│/∑_*hkl*_│*F*_o_│ for all data with *F*_o_ *>*2σ(*F*_o_), excluding data used to calculate *R*_free_. §*R*_free_ =∑_*hkl*_│*F*_o_-*F*_c_│/∑_*hkl*_│*F*_o_│ for all data with *F*_o_ *>*2σ(*F*_o_) that were excluded from refinement

### Isothermal titration calorimetry

Isothermal titration calorimetry (ITC) measurements were performed on a Microcal iTC 200 (GE Healthcare) VP-ITC microcalorimeter at 298 K. The protein was dialyzed against 20 mM Hepes (pH 7.0) and 150 mM NaCl. The titration CdCl_2_ solution was prepared with 20 mM Hepes (pH 7.0) and 150 mM NaCl by adding 2 mM CdCl_2_. Both the protein and the titrant CdCl_2_ solutions were thoroughly degassed in a ThermoVac apparatus (Microcal). The titration reaction was performed by sequential injections of 40 μl CdCl_2_ solution into the sample cell. The duration of the injection was 120 s. The syringe was rotated at 600 rev min^-1^. Triplet measurements were collected in each case.

### Kinase-Glo® luminescent kinase assay

The *in vitro* ATPase activity of UspE was measured by quantifying the amount of ATP remaining in the solution following a kinase reaction using a Kinase-Glo® Luminescent Kinase Assay Kit (Promega, Fitchburg, WI, USA). The assay was performed in a 96-well plate in a kinase reaction volume of 50 μl containing 10 mM MgCl_2_,5 μM ATP,10 mM HEPES (pH 8.0) and 150 mM NaCl. The reaction was initiated by adding the protein to a final concentration of 0.4 mg/ml-3.2 mg/ml. The reaction mixture was kept at 310 K for 20 min in a water bath. Reaction mixtures containing no UspE were used as negative controls. The kinase reaction mixture was incubated with 50 μl of ATP detection reagent. The plates were then incubated for another 10 min at 310 K. The Synergy2 Multi-Mode Microplate Reader (BioTek, Winooski, VT, USA) was used to collect the relative light unit (RLU) signal. The luminescent signal was positively correlated with the amount of remaining ATP and inversely correlated with the amount of kinase activity.

## Results and discussion

### Overall structure

**Fig. 1:**
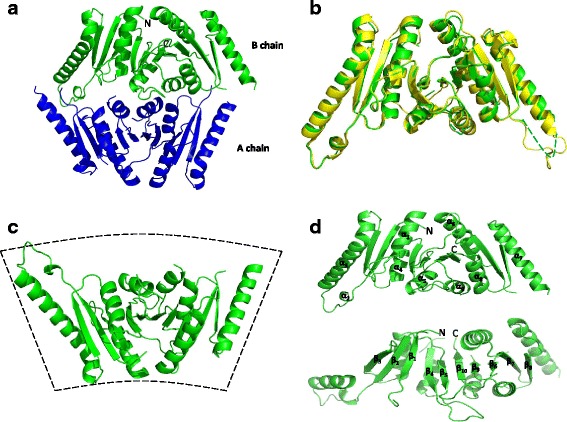
Overall structure of the UspE. **a** Structure of *Ec*UspE in the tetragonal crystal form, displayed as ribbons. The asymmetric unit contains two protomers colored blue and green. **b** Structural comparison of the cartoon traces of *Ec*UspE and *P. mirabilis* USP (PDB code: 4WY2). *Ec*UspE is colored green, and the *P. mirabilis* USP is colored yellow. The disordered regions are shown with dashed lines. **c** The monomer structure of *Ec*UspE. **d** Secondary structural elements of UspE are numbered

To examine the biochemical mechanisms responsible for UspE function, we determined the crystal structure of the UspE by the molecular replacement method with the synchrotron data set at a resolution of 3.2 Å. The final model refined to a R-factor of 0.24 (R_free_ = 0.30). The initial solution suggested the presence of two monomers per asymmetric unit, which is consistent with the Matthews’ coefficient of 3.1 Å^3^ Da^-1^ (60.37 % solvent). The tertiary structure of the UspE is very similar to that of the previously described *P. mirabilis* Usp protein PMI1202 (PDB code: 3OLQ), which was used as a search model in molecular replacement [13, 14]. UspE exists as a monomer, and the structure reveals a compact and 2-fold symmetric dimer in the crystal. Each monomer consists of two USP domains, and the final model contains two homologous subunits related by a non-crystallographic symmetry (Fig. 1a). The *E. coli* UspE has a high structural similarity compared with the *P. mirabilis* USP (PDB code: 4WY2) (Fig. 1b). The crystal structure shows that UspE is folded into a fan-shaped structure similar to that of the tandem-type Usp protein USP from *P. mirabilis* (Fig. 1c)*.* In a Ramachandran plot, 93.82 % of the model residues were found in favored regions, 5.72 % in allowed regions, and 1.06 % in the disallowed regions. Their structures are virtually identical with a root-mean-square deviation (RMSD) value of 0.40 Å for a 244 Cα atom. UspE is composed of ten β-stranded mixed β-sheet and nine α-helices. In the core structure, ten β-strands form a central parallel β-sheet (Fig. 1d). Significant density was observed for all residues in the final electron density map except 163–170, 202–215, and 270–282. Table 1 provides refinement statistics and structure solution for all structures.

### *E. coli* UspE accommodates an unidentified ligand

**Fig. 2:**
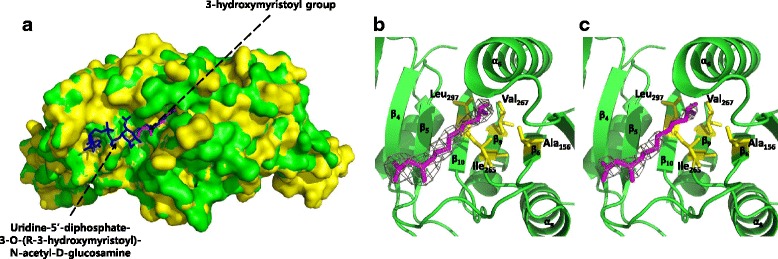
Unidentified ligand bound in the crystal structure of UspE. **a** Structural comparison of the surface traces of *Ec*UspE and *P. mirabilis* USP (PDB ID: 4WY2). *Ec*UspE is colored green, and the *P. mirabilis* USP is colored yellow. Uridine-5′-diphosphate-3-O-(R-3-hydroxymyristoyl)-N-acetyl-D-glucosamine and 3-hydroxymyristoyl group are colored magentas and yellow, respectively. **b** The 2*F*_o_ - *F*_c_ map around the ligand is contoured at the 1 sigma level (gray). (C) The 2*F*_o_ - *F*_c_ map around the ligand is contoured at the 2 sigma level (gray)

More importantly, we found an unambiguous stick-like electron density in the hydrophobic pocket of *E. coli* UspE throughout the refinement process. It looks like that the UspE carries the unidentified ligand. The crystal structure of *P. mirabilis* USP suggested that Uridine-5′-diphosphate-3-O-(R-3-hydroxymyristoyl)-N-acetyl-D-glucosamine was tightly bound to *P. mirabilis* USP. We found that the Uridine-5′-diphosphate-3-O-(R-3-hydroxymyristoyl)-N-acetyl-D-glucosamine binding pocket of *P. mirabilis* USP was very similar to the hydrophobic pocket of *E. coli* UspE (Fig. 2a) and that the hydrophobic pocket of *E. coli* UspE was appropriate for binding Uridine-5′-diphosphate-3-O-(R-3-hydroxymyristoyl)-N-acetyl-D-glucosamine. In addition, the stick-like electron densities were very similar to a 3-hydroxymyristoyl group of Uridine-5′-diphosphate-3-O-(R-3-hydroxymyristoyl)-N-acetyl-D-glucosamine. Thus, this group was placed at density result. Despite the fact that *Ec*UspE crystals were obtained in the absence of 3-hydroxymyristoyl group molecules in both media and buffers, these positions could be successfully refined with no significant residual difference density and with associated B-factors comparable with those of the surrounding atoms. The characteristic hydrophobic environment in the pocket indicates that UspE can bind unidentified ligand with a 3-hydroxymyristoyl group in the cavity. This pocket was surrounded by hydrophobic residues in β6, β9, β10, and α6 (Fig. 2b and c). This finding indicates that hydrophobic interactions are involved in the binding of this ligand. A previous study using mass spectrometric and surface analyses showed that the UspE homologue protein YdaA from *Salmonella enterica* serovar Typhimurium might bind a large, non-polar ligand in its N-terminal domain; however, YdaA was not bound to any ligand in crystal structure [15]. The ligand bound to *P. mirabilis* USP was tentatively identified as UDP-(3-O-(R-3-hydroxymyristoyl))-N-acetylglucosamine. The published crystal structures of the LpxA and LpxC from *E. coli* contain UDP-(3-O-(R-3-hydroxymyristoyl))-N-acetylglucosamine and its deacetylated product, respectively [16, 17]. These proteins catalyze the first committed step of lipid A biosynthesis [18]. Combined with these data, the unidentified ligand bound to *E. coli* UspE is probably related to an intermediate in lipid A biosynthesis, similar to UDP-(3-O-(R-3-hydroxymyristoyl))-N-acetylglucosamine deacetylases.

### *E. coli* UspE has an ATP binding motif and ATPase activity

**Fig. 3:**
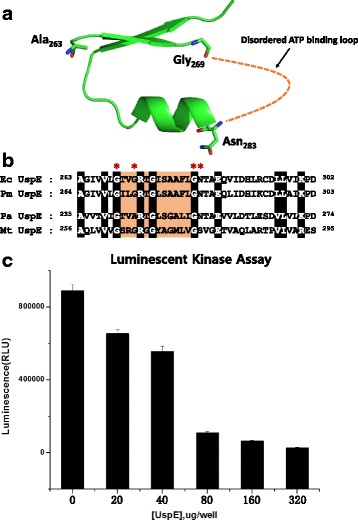
ATP binding motif and ATPase activity of UspE. **a** Close-up view of the **b** Alignment of the sequences around the ATP binding motif from *E. coli* UspE (Ec UspE), *P. mirabilis* (Pm UspE), *P. aeruginosa* UspE (Pa UspE), and *M. tuberculosis* (Mt UspE). The conserved residues and ATP binding motif are highlighted. **c** Kinase activity of UspE. Varying concentrations (0.4, 0.8, 1.6, 3.2, 6.4, mg/ml) of UspE were used in the reaction mixture, and the protein are resuspended in 50 μl containing 10 mM MgCl_2_ and 5 μM ATP. The graph represents the mean of three independent experiments, and the standard deviation is indicated by error bars

Previous studies show that USPs can be divided into groups: those that bind ATP (UspFG-type), those that do not bind ATP (UspAs and UspA-like group), and those that hydrolyze adenine nucleotide substrates. USPs that bind ATP may function as an ATP-dependent signaling intermediate in a pathway that promotes persistent infection. Furthermore, it was suggested that USPs contain a conserved-sequence Gly-X_2_-Gly-X_9_-Gly (Ser/Thr/Asn) motif that is needed for binding ATP [3, 5, 6, 15, 19, 20]. However, the ATP binding motif of UspE was disordered in the crystal, due to the intrinsic flexibility of these regions (Fig. 3a). Therefore, it is likely that the bound ATP is disordered or the protein devoid of bound ATP was preferentially crystallized. In this structure, we found the presence of this motif (Gly^269^- X_2_-Gly^272^-X_9_-Gly^282^-Asn) in the C-terminal domain of UspE, which is similar to other USPs that bind ATP (Fig. 3b). The *in vitro* ATPase activity of UspE was determined by measuring the amount of ATP left in solution following a kinase reaction using the Kinase-Glo® Luminescent Kinase Assay. As expected, the decline in luminescent signal depended on the increasing concentration of UspE (Fig. 3c). These results indicate that ATPase activity from UspE decreases the remaining ATP levels.

### UspE has putative Cd^2+^ binding sites

**Fig. 4:**
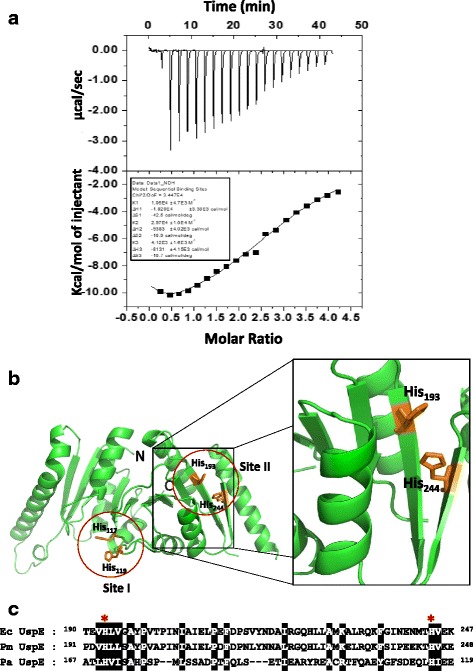
Representative isotherm for the binding of Cd^2+^ to UspE. **a** In each panel, top: raw data output of power (heat released) for each of 25 consecutive injections of CdCl_2_ (2 mM) or mitomycin C (2 mM) in to the protein (0.2 μM). Bottom: heat exchange at each injection obtained by integration of each injection, normalized to kcal/mol of CdCl_2_. The computer generated titration curve is best fit to a model of sequential binding with three sites (solid line). **b** The Cd^2+^ binding site I and site II are shown in the red circle. Close-up view of the Cd^2+^ binding site II, His193 and His 244 is shown as sticks colored orange. **c** Sequence alignment of the Cd^2+^ binding site II part of UspE proteins. The key conserved regions are highlighted in black. In key conserved regions, His 193 and His 244 are denoted with asterisks

Cadmium, in a variety of chemical forms, is toxic for the proper growth of microbial cells. Previous studies showed that cadmium (273 μM) can cause complete but transient inhibition of growth accompanied by the synthesis of cadmium-induced proteins (CDPs) [21]. The *E. coli* increase synthesis of CDPs (e.g., H-NS, UspA, UspC, UspD, UspE), which together make up the cadmium stress stimulon [7]. The UspE can sequester Cd from the cytosol to protect themselves. To analyze the relationship between UspE and cadmium, we investigated the cadmium binding ability of UspE by an ITC experiment. The ITC experiment was carried out using cadmium as titrant at pH 7.0. Initial attempts to fit the data to a double site-binding model were not successful. The isotherm was best fit when a sequential binding model with three binding sites was applied. Through analysis of these ITC data, we observed tight Cd^2+^ binding to UspE with *n* = 3. This suggests a binding stoichiometry of 3 moles of Cd^2+^ to 1 mole of UspE. The two have moderate binding affinities (K_d_ of 33.7 and 94.3 μM, respectively), whereas the other one has low affinity (K_d_ of 242.7 μM). The K_d_ value of site 1 and site 2 are high compared with site 3. At site 1 and site 2, the binding of Cd^2+^ with UspE is favorable, with an exothermic enthalpy (∆H of -18.2 and -9.384 kcal mol^-1^, respectively) and negative entropy (∆S = -12.67 and -3.25 kcal mol^-1^, respectively). At site 3, the binding of Cd^2+^ with UspE is unfavorable, with an exothermic enthalpy (∆H = -8.131 kcal mol^-1^) and negative entropy (∆S = -3.19 kcal mol^-1^). The detailed thermodynamic parameters are listed in Table 2 and Fig. 4a. Evidence has been reported that to obtain lethal effects in an exponentially growing culture, 600 μM CdCl_2_ is required. In the lag phase before growth commenced, 3 μM CdCl_2_ inhibited cell proliferation and 10 μM was lethal [13, 14]. To regulate the concentration of Cd^2+^, *E. coli* might induce CDPs such as UspE. These results suggest that apoUspE and Cd^2+^ have a direct relationship. To understand the relationship between apoUspE and Cd^2+^, we observed the ability of cadmium to interact with UspE through the ITC experiment. Our structural analysis indicates that *E. coli* has two putative binding sites (Site I: His117, His 119; Site II: His193, His244) (Fig. 4b). Additionally, sequence alignment showed that residues His117, His119, His193, and His244 within the β-barrel domain are highly conserved among the UspE proteins (Fig. 4c). Recently, a study has been performed to observe site I, which is known to be crucial for zinc binding in the crystal structure of YdaA from *S. enterica* serovar Typhimurim (PDB ID: 4R2J) [15]. This provides further support for the original conclusion that cadmium binds at two locations. Our results clearly demonstrate that *E. coli* UspE has two different sets of binding sites and the protein may provide additional confirmation for the cadmium binding to these two sites.

**Table 2:** ITC experiment Cd^2+^ binding to UspE

	Kd	ΔH	TΔS	ΔG
	μM	kcal/mol	kcal/mol	kcal/mol
Site 1	94.3	-18.2	-12.67	-5.5
Site 2	33.7	-9.383	-3.25	-6.1
Site 3	242.7	-8.131	-3.19	-5.0

In summary, the crystal structure of UspE from *E. coli* is representative of a tandem-type USP. The crystal structure of *E. coli* UspE reveals a hydrophobic pocket that moderately binds an unidentified ligand. Combined with previous studies, we can conclude that UspE is probably related to an intermediate in lipid A biosynthesis. We subsequently found through the sequence analysis that UspE has an ATP binding motif (Gly^269^- X_2_-Gly^272^-X_9_-Gly^282^-Asn) in the C-terminal domain and has ATPase activity, though this did not appear in the crystal structure. We were also able to perform an ITC experiment which revealed that UspE probably has two Cd^2+^ binding sites and that the His117, His119, His193, and His244 residues within the β-barrel domain are critical for binding Cd^2+^. We believe that this information is a significant contribution to understanding the molecular mechanisms of *E. coli* UspE.

## Conclusions

In this study, we have determined the crystal structure of UspE of *E. coli* as a representative of a tandem-type USP. The UspE consists of two tandem USP domains that are highly conserved in this protein family. We found a hydrophobic pocket in the UspE structure, which was strongly bound to unidentified ligand. Combined with a previous study, evidence suggests that the UspE is related to an intermediate in lipid A biosynthesis. We subsequently found that sequence analysis suggests that UspE has an ATP binding motif (Gly^269^- X_2_-Gly^272^-X_9_-Gly^282^-Asn) in the C-terminal domain of UspE and has ATPase activity, but this was not confirmed by the crystal structure. We were also able to perform the ITC experiment, which revealed that the UspE probably has two Cd^2+^ binding sites, comprised of the His117, His 119, His193, and His244 residues within the β-barrel domain. Both of them are essential for Cd^2+^ binding to UspE protein. As discussed before, USPs might be associated with several functions, such as cadmium binding, ATPase activity, and an intermediate in lipid A biosynthesis.

## Abbreviations

*E. coli*: *Escherichia coli*
ITC: isothermal titration calorimetry
MR: molecular replacement
*P. mirabilis*: *Proteus mirabilis*
PDB: protein data bank
RLU: relative light unit
RMSD: root-mean-square deviation
*S. enterica* serovar Typhimurim: *Salmonella enterica* serovar Typhimurium
USP: universal stress protein
